# Efficacy and safety of Cadonilimab plus anlotinib in small cell lung cancer with brain metastases

**DOI:** 10.3389/fonc.2025.1545101

**Published:** 2025-07-17

**Authors:** Hai-Zhen Yi, Wei Lv, Jin-Jing Chen, Zhan Lin

**Affiliations:** ^1^ Department of Medical Oncology, The First People’s Hospital of Yulin, Yulin, Guangxi, China; ^2^ Department of Medical Center, The First People’s Hospital of Yulin, Yulin, Guangxi, China; ^3^ Department of Medical Hematology, The First People's Hospital of Yulin, Yulin, Guangxi, China

**Keywords:** small cell lung cancer, Cadonilimab, brain metastases, PD-1, anlotinib

## Abstract

**Purpose:**

This study aimed to evaluate the efficiency and safety of Cadonilimab and Anlotinib pairing in individuals diagnosed with small cell lung cancer (SCLC) and brain metastases (BMs).

**Methods:**

A review was performed on individuals who were diagnosed with small cell lung cancer (SCLC) and had central nervous system (CNS) metastases confirmed via magnetic resonance imaging (MRI) of the brain.We assessed the treatment response of Cadonilimab plus Anlotinib using Response Evaluation Criteria in Solid Tumors version 1.1 (RECIST 1.1) and Response assessment in neuro-oncology brain metastases (RANO-BM) for evaluating solid tumors and neuro-oncology brain metastases, respectively.The patients’ prognosis was determined using Kaplan-Meier analysis and Cox regression analysis.

**Findings:**

The study initially included 46 patients diagnosed with SCLC who presented with brain metastases (BMs). According to the RANO-BM criteria, intracranial lesions showed an objective response rate (ORR) of 41.3%. The median overall survival (OS) was observed to be 19.3 months (95% CI, 17.4-21.1 months). Multivariate Cox regression analysis showed that having a PD1 level below 50% (HR=4.83, *P <*0.001) or having two or more metastatic organs (HR = 2.71, *P* = 0.036) were independent factors that positively predicted overall survival of all the patients, 86.9% experienced treatment-related adverse events (TRAEs) associated with the treatment, while 17.4% reported severe TRAEs of grade3-4.

**Implications:**

According to our results, the combination of Cadonilimab and Anlotinib appears to be a promising treatment option for SCLC patients with brain metastases.

## Introduction

Approximately 15% of patients newly diagnosed with lung cancer have small cell lung cancer (SCLC), which is associated with high rates of recurrence and mortality (1). Furthermore, individuals afflicted with this fatal illness are at a higher risk of developing brain metastases (BMs) and have limited treatment alternatives, leading to an abnormal survival rate. The median overall survival (OS) of patients with small cell lung cancer (SCLC) typically spans from 3.2 to 8.5 months, with nearly half of these patients developing brain metastases (BMs) during their treatment (2). It is currently recommended that radiotherapy take the form of stereotactic radiation (SRS) or whole-brain radiation (WBRT) in combination with systemic therapy for the treatment of metastatic SCLC patients with BMs (3). Owing to the cognitive decline that often accompanies treatment, along with the high relapse rate, patients with asymptomatic and stable BMs should exercise caution when considering brain radiotherapy, as it may cause more harm than good. Conversely, traditional chemotherapy is not very effective because of the presence of the blood-brain barrier (BBB).

Advanced cancer treatment has been revolutionized by the utilization of immune checkpoint inhibitors (ICIs), which tap into the potential of the immune system. Additionally, extensive-stage SCLC patients could expect a significantly better prognosis with ICIs.Liu, and Liu SV and Rudin CM have demonstrated that the inclusion of PD-L1 inhibitor (atezolizumab) and PD-1 inhibitor (pembrolizumab) in chemotherapy can significantly extend overall survival (OS) compared to conventional chemotherapy (atezolizumab [HR=0.70, 95%CI 0.54–0.91, P=0.007] and pembrolizumab [HR=0.80, 95%CI 0.64–0.98, P=0.0164]) (4, 5). However, subgroup analyses of these randomized trials (KEYNOTE-604, HR =1.07, 95%CI 0.60–1.91; IMpower133, HR=0.96, 95% CI 0.46–2.01) did not show that ICIs combined with chemotherapy have any benefit for BMs SCLC patients. Advanced cancer treatment has been revolutionized by the utilization of immune checkpoint inhibitors (ICIs), which tap into the potential of the immune system. Additionally, extensive-stage SCLC patients could expect a significantly better prognosis with ICIs.Liu, and Liu SV and Rudin CM have demonstrated that the inclusion of PD-L1 inhibitor (atezolizumab) and PD-1 inhibitor (pembrolizumab) in chemotherapy can significantly extend overall survival (OS) compared to conventional chemotherapy (atezolizumab [HR=0.70, 95%CI 0.54–0.91, P=0.007] and pembrolizumab [HR=0.80, 95%CI 0.64–0.98, P=0.0164]) (4, 5). However, subgroup analyses of these randomized trials (KEYNOTE-604, HR =1.07, 95%CI 0.60–1.91; IMpower133, HR=0.96, 95% CI 0.46–2.01) did not show that ICIs combined with chemotherapy have any benefit for BMs SCLC patients. Therefore, the development of novel therapeutic strategies is necessary.

Cadonilimab is an IgG1 antibody with a tetravalent bispecific structure and includes a single-chain variable fragment (scFv). The Fc-null configuration eliminates antibody-dependent cytotoxicity (ADCC), antibody-dependent phagocytosis (ADCP), complement-dependent cytotoxicity (CDC), and cytokine generation. Fc receptor-mediated effector functions, which eliminate or harm PD-1 and cytotoxic T-lymphocyte antigen-4 (CTLA-4) expressing lymphocytes, may diminish antitumor efficacy. Cadonilimab exhibits notably reduced toxicities compared with alternative medications in clinical settings. Within a tumor-like environment, the strong affinity of Cadonilimab and its Fc-null structure might enhance drug retention and safety, ultimately leading to effective antitumor outcomes (6, 7). According to researchers, the combination of chemotherapy with anlotinib, a new multitargeted tyrosine kinase inhibitor (TKI), enhances progression-free survival and quality of life in SCLC patients with BMs (7, 8). Numerous studies have demonstrated the synergistic effects of immunotherapy and antiangiogenic therapy in advanced solid tumors. Clinical studies of anti-PD-1/PD-L1 antibodies in combination with anti-angiogenic drugs have shown promising efficacy and manageable safety in treating SCLC. Preclinical studies investigating synergies with ICIs suggest that anlotinib has the potential to modify the microenvironment of tumor immunity by suppressing the expression of PD-L1 on endothelial cells in the vascular system and facilitating the infiltration of cells from the innate immune system. Anlotinib, a new orally administered tyrosine kinase inhibitor that targets vascular endothelial, fibroblast, and platelet-derived growth factor receptors, has been approved as a third-line therapy for advanced SCLC in China, However, these studies did not targeted in these studies. In our study, the combination of Anlotinib with Cadonilimab was evaluated as a potential treatment for advanced SCLC patients with BMs.

## Patients and methods

### Study design

Therefore, this study retrospectively screened advanced-stage small cell lung cancer (SCLC) patients with brain metastases (BMs). who received anlotinib plus cadonilimab in clinical practice from June 2019 to December 2022 at The First People’s Hospital of Yulin. included the following inclusion criteria: (1) SCLC diagnosis by pathology or cytology, with extensive‐stage SCLC (ES‐SCLC); (2) age ≥ 18 years; (3) Magnetic resonance imaging of the brain (magnetic resonance imaging, MRI) contrasted with baseline BMs; (4) patients who had previously undergone systemic therapy but had not been treated with the combination of ICIs and Anlotinib, nor with ICIs or Anlotinib as standalone therapies;not receive Prior intracranial RT=0; (5) Eastern Cooperative Oncology Group (ECOG) performance status of 0–2 score; (6) measurable target lesions according to RECISTv1.1 criteria to assess the therapeutic response. The exclusion criteria were as follows: (1) diagnosis of one more tumor or a serious disease that might compromise their life; (2) patients receiving systemic treatment other than anlotinib combined with cadonilimab; (3) efficacy assessment data were not available;(4) patients who had received combination therapy before developing BMs or did not have baseline imaging and at least one follow-up scan were not included in the study. The study complied with the Declaration of Helsinki and the International Good Clinical Practice Guidelines. The Institutional Review Board approved the waiver of written informed consent (9–11).

### Endpoints of the study

The data were collected and analyzed using Response Evaluation Criteria in Solid Tumors version 1.1 (RECIST 1.1) and Response Assessment in Neurooncology Brain Metastases (RANO-BM). Tumors with a diameter ≥ 10 mm were considered measurable lesions, which were classified into four categories: complete response (CR), partial response (PR), stable disease (SD), and progressive disease (PD). the objective response rate (ORR), which represents the ratio of systemic objective responses, was determined by calculating the percentage of extracranial lesions showing complete or partial response. The DCR or disease control rate was calculated by considering complete response, partial response, and stable disease. Intracranial ORRs and DCRs were calculated according to brain lesions. The OS period was defined as the period from the start of treatment to the date of death or the date of last imaging. Progression-free survival (PFS) refers to the duration from treatment initiation to the occurrence of intracranial or extracranial progression, death, or censoring at the time of the most recent imaging. Progression-free survival (PFS) of the brain tumor was determined from From the onset of treatment until the end, mortality, or censoring at the final imaging assessment. Patients who experienced extracranial progression first were not included in the calculation of intracranial PFS. Adverse events associated with treatment TRAEs (treatment-related adverse events) were documented throughout the course of the treatment and subsequent monitoring.

### Statistical analysis

Descriptive summaries were used to analyze clinical and demographic variables, and Kaplan-Meier curves for overall survival (OS) and progression-free survival (PFS) were generated. Censored observations were considered for analyses when patients did not experience disease progression or when they reached the end of follow-up. Clinical characteristics were compared using the chi-squared test. Log-rank tests were used to compare survival between different groups, and we investigated the correlation between survival and clinical factors using both univariate and Multivariable Cox regressions. SPSS18 was used for all analyses, was the consideration for *P* < 0.05.

## Results

**Figure 1:**
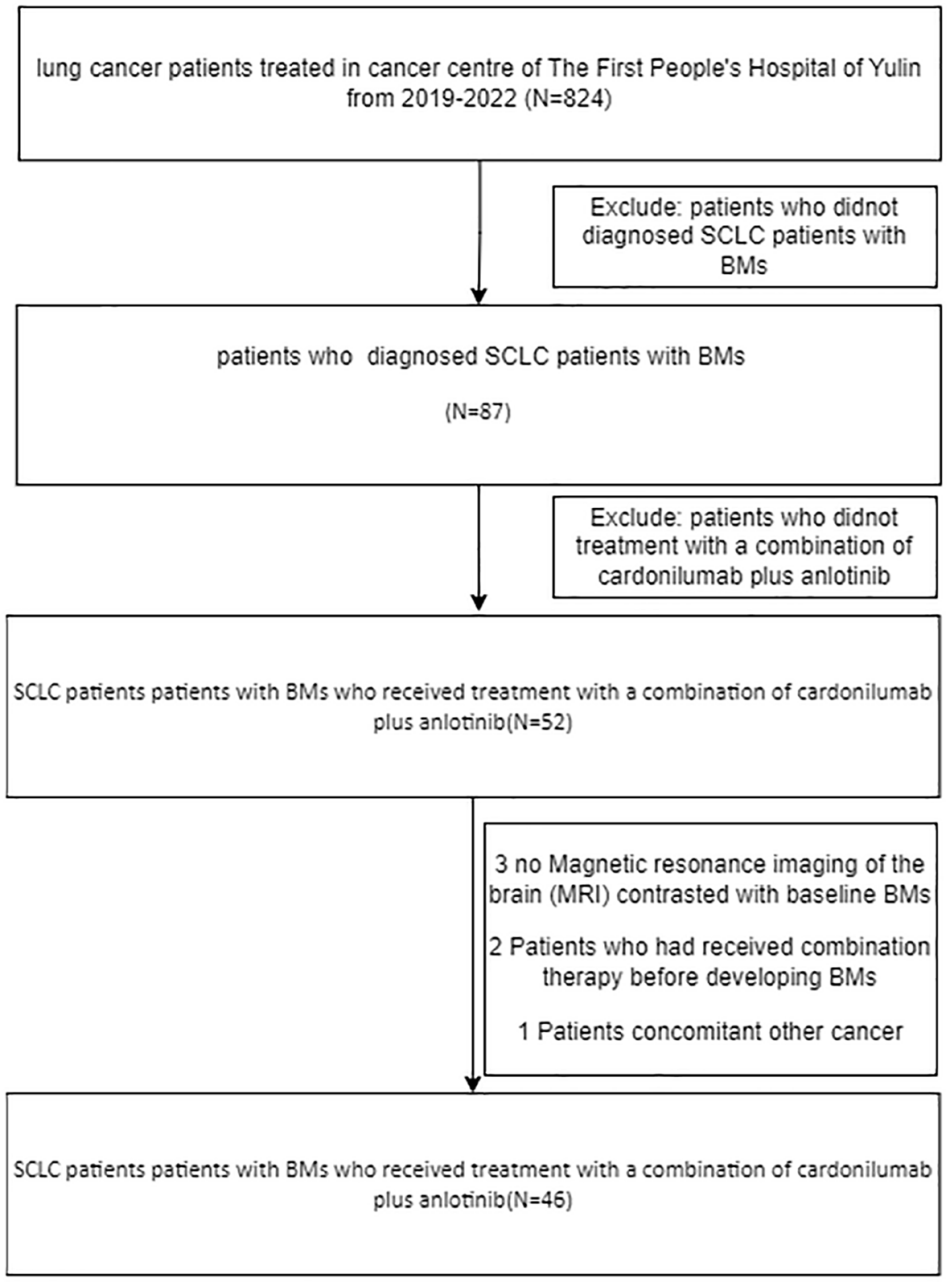
Flowchart of the study design. SCLC, small cell lung cancer.

The study profile is illustrated in **Figure 1**, A total of 46 patients with advanced small cell lung cancer (SCLC) and brain metastases (BMs) received treatment with a combination of Cadonilimab plus Anlotinib. **Table 1** summarizes the fundamental clinical and pathological characteristics of these patients. The majority of the patients (approximately 65.2%) were below the age of 60 and exhibited an Eastern Cooperative Oncology Group (ECOG) performance status of 0-1, which accounted for 87% of the total.32.6% of patients had metastatic disease affecting more than two organs. Of the total number of patients, 20 (43.5%) experienced brain lesions with symptoms, while 34 patients (73.9%) had multiple brain lesions. Additionally, 63.1% of the subjects attained lactate dehydrogenase levels(LDH) surpassing the upper limit of normal (ULN).36.9% of patients achieved higher LDH levels than the ULN.

**Table 1:** Baseline characteristics of the study population.

Characteristic	N (%)
Age	
< 65	30 (65.2%)
≥ 65	16 (34.8%)
Sex	
Male	38 (82.6%)
Female	8 (17.4%)
ECOG-PS	
0–1	40 (87%)
2	6 (13%)
Smoking history	
Never	28 (60.8%)
Smoked	18 (39.2%)
Metastatic organs	
2	31 (67.4%)
≥ 2	15 (32.6%)
Number of BMs	
Single	12 (26.1%)
Multiple	34 (73.9%)
Symptomatic BMs	
No	26 (56.5%)
Yes	20 (43.5%)
Prior lines of systemic therapy	
2	11 (23.9%)
≥3	37 (80.4%)
Prior intracranial RT	
No	46
Yes	0
Prior ICIs treatment	
No	46
Yes	0
Concurrent intracranial RT	
No	46
Yes	0
LDH	
< ULN	29 (63.1%)
≥ ULN	17 (36.9%)
PD1/PDL-1	
>50%	21
<50%	25

### Evaluating effectiveness

**Figure 2:**
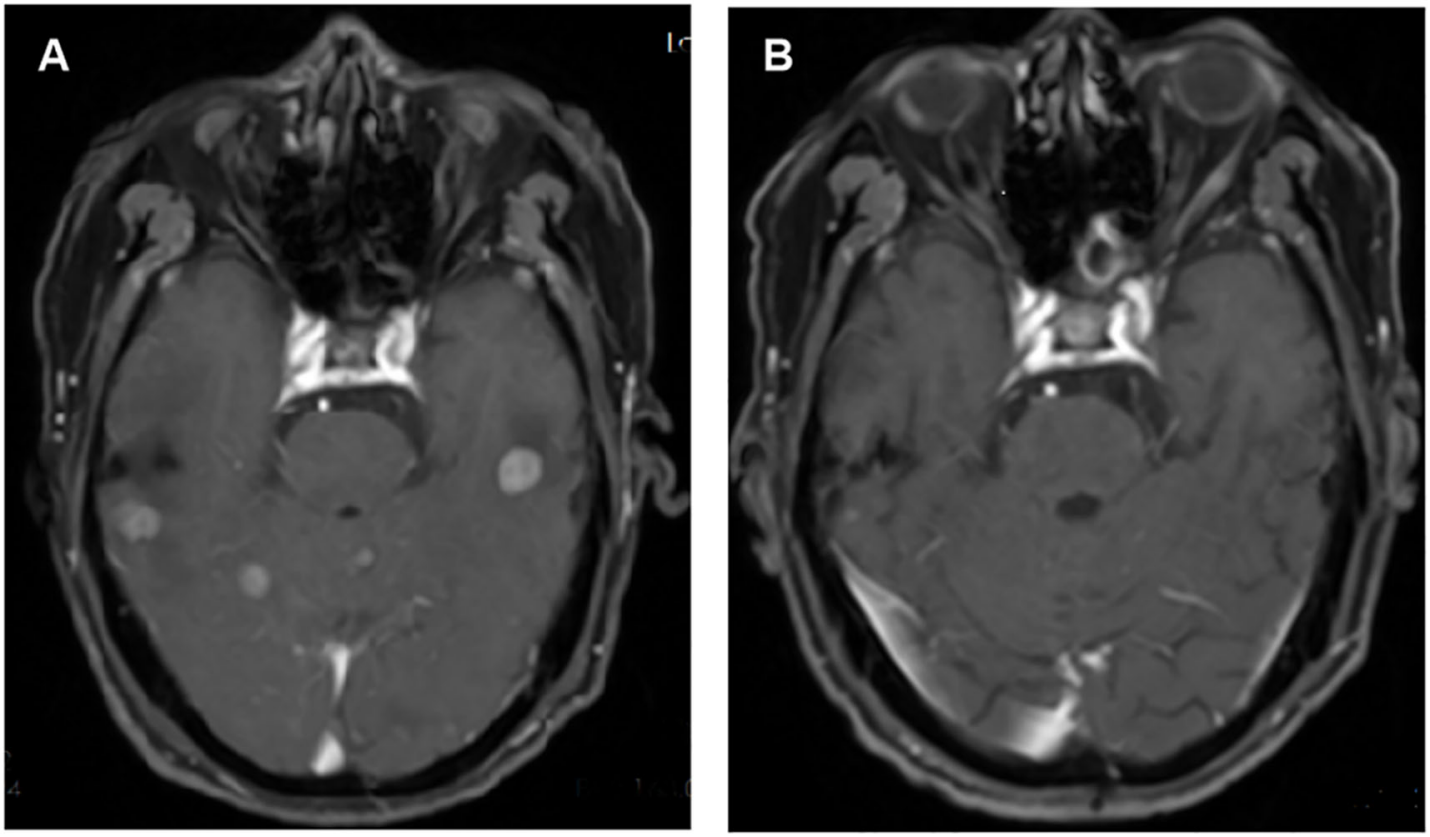
**(A)** Pre-treatment and **(B)** post-treatment of the intracranial lesion in weighted magnetic resonance images.

**Figure 3:**
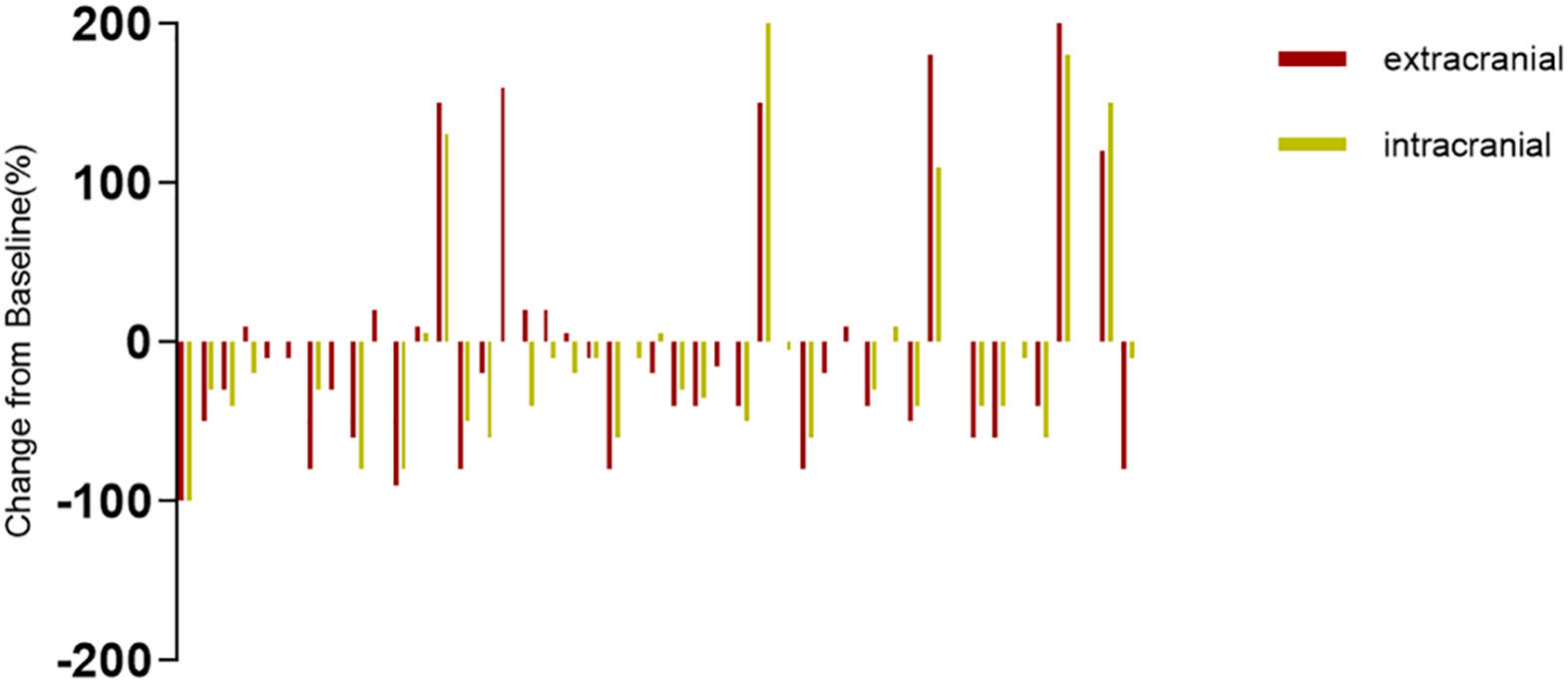
The intracranial and extracranial changes in SCLC patients with measurable intracranial lesions are presented in the bar graph.

**Figure 4:**
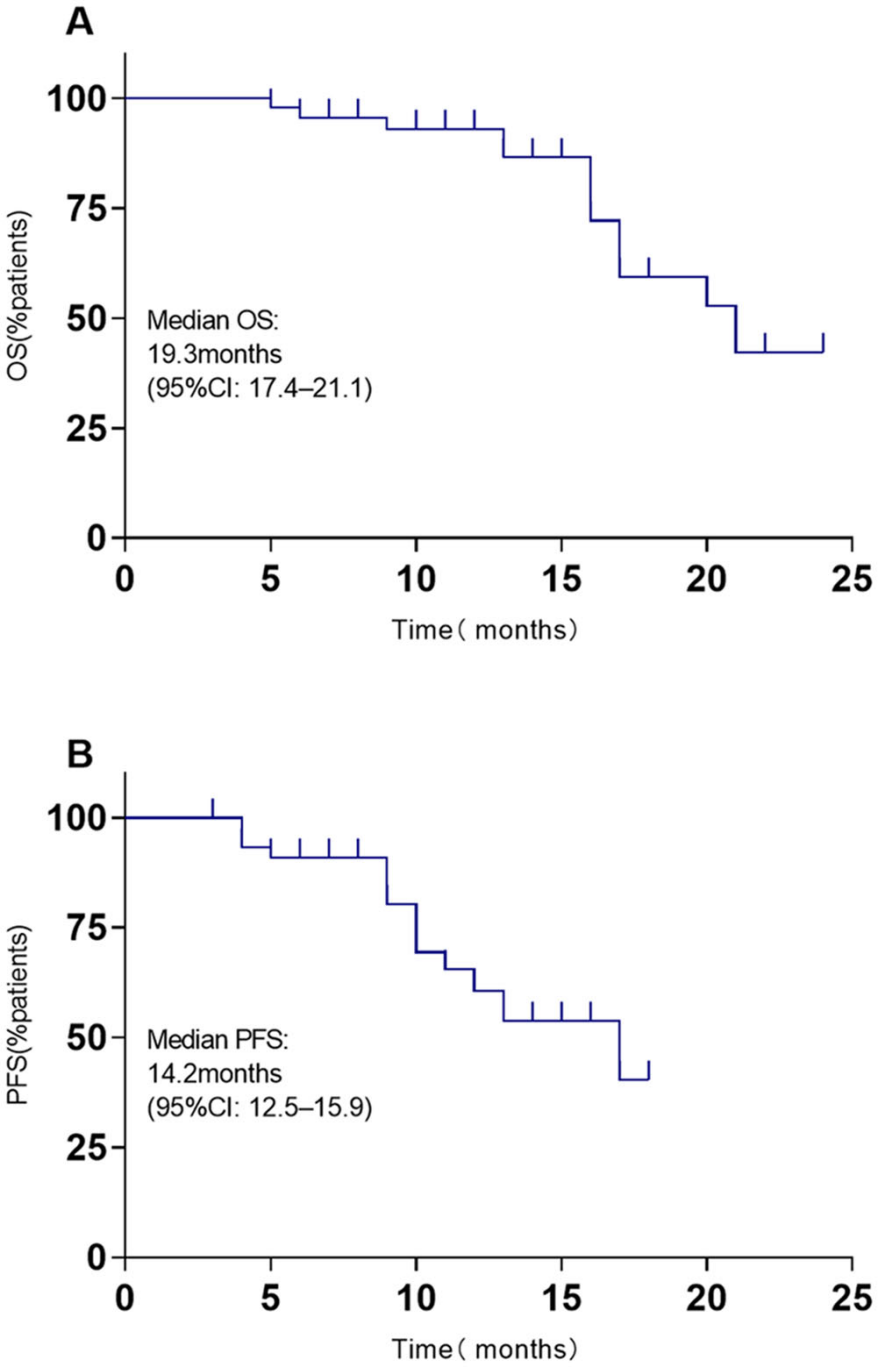
OS and PFS based on Kaplan-Meier survival analysis. **(A)** OS; **(B)** PFS.

The systemic responses of all 46 patients were assessed. The overall response rate (ORR) and disease control rate (DCR) for these patients were 45.7% and 86.9%, respectively (**Table 2**). It contains a summary of the treatment response to Cadonilimab plus Anlotinib, evaluated using RECIST 1.1 criteria.RANO-BM criteria were also used to assess intracranial response, which was 41.3% (1 CR, 18 PR) (**Table 2**). **Figure 2** shows typical MRI scans of a complete response in brain lesions before and after therapy; patients with detectable lesions are depicted in **Figure 3**, illustrating alterations both inside and outside the skull. In the overall study, In the overall study population, the median overall survival (OS) was found to be 19.3 months(95% CI, 17.4–21.1 months), and the median progression-free survival (PFS) was 14.2 months(95% CI, 12.5–15.9 months) (**Figures 4A, B**).

**Table 2:** Efficacy of Cadonilimab plus Anlotinib.

Characteristic	N (%) (N = 46)
Response to Cadonilimab plus Anlotinib treatment, measured using the RECIST 1.1 criteria.	
CR	1
PR	20
SD	19
PD	6
ORR	21 (45.6%)
DCR	40 (86.9%)
The intracranial efficacy of Cadonilimab plus Anlotinib was evaluated in patients with measurable lesions per RANO-BM.	
Reduction of steroid treatment	
No	10
Yes	36
Neurological symptoms	
Stable or improved	44
Worse	2
T2/FLAIR signal	
Stable or decreased	42
Increased	4
Intracranial response	
CR	1
PR	18
SD	23
PD	4
ORR	19 (41.3%)
DCR	42 (91.3%)

### Prognosis

**Figure 5:**
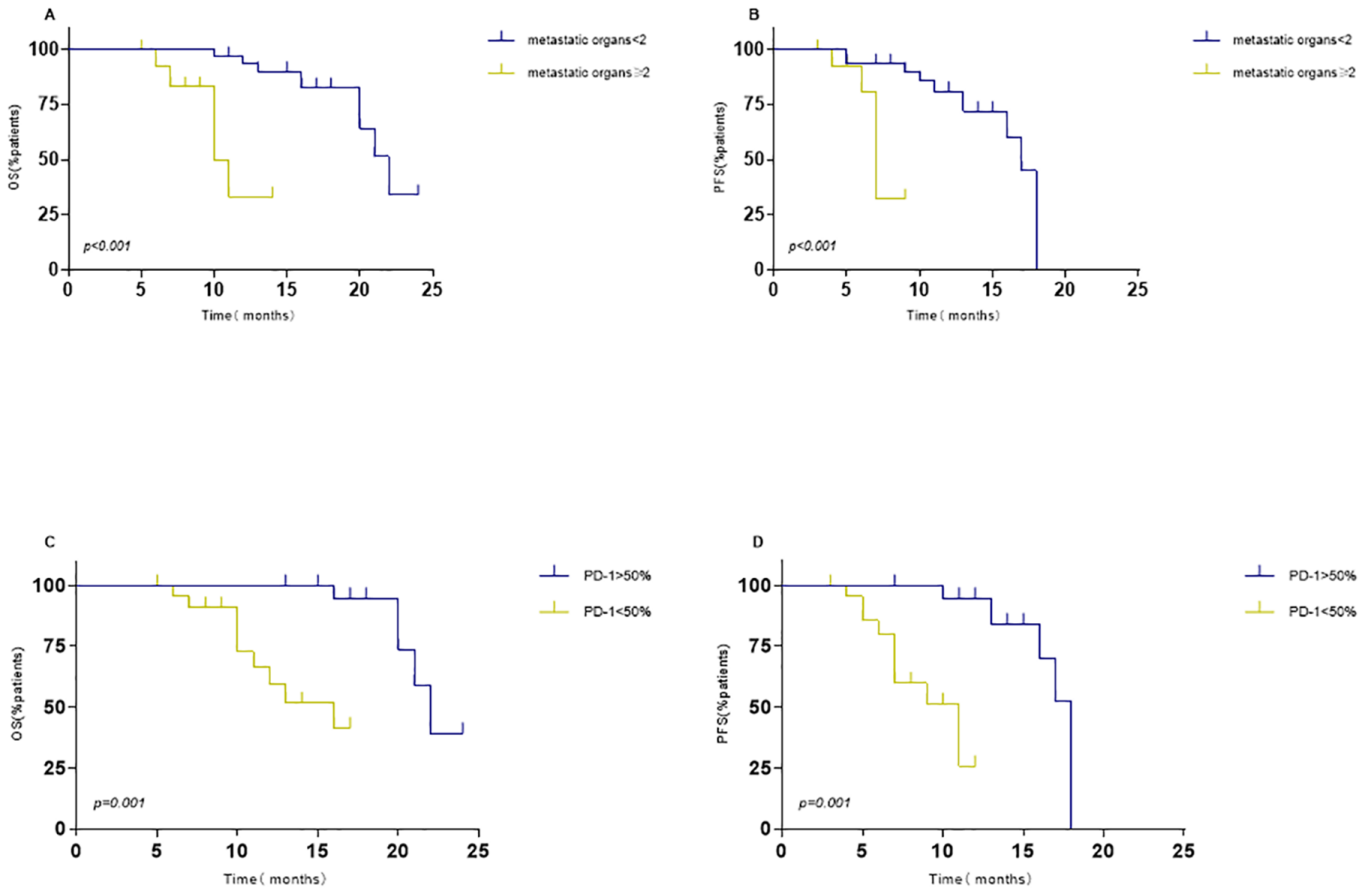
Kaplan–Meier survival analysis for overall survival (OS) and progression-free survival (PFS) based on significant predictors. OS: **(A)** metastatic organs ≥2 vs. without metastatic organs ≥2; **(C)** PD1>50% vs. PD1<50%. PFS: **(B)** metastatic organs ≥2 vs. without metastatic organs ≥2; **(D)** PD1>50% vs. PD1<50%.

In addition, we investigated the impact of various factors on survival using univariate and multivariate Cox models. The multivariate analysis encompassed all variables that showed significant correlations and trends (*P* < 0.05) in the univariate analysis. Multivariable Cox model analysis for overall survival (OS) included smoking, number of brain metastases (BMs), and number of metastatic organs. PD1 < 50% (HR = 4.83, *P*< 0.001) or metastatic organs ≥ 2 (HR = 2.71, *P* = 0.036) were independent positive predictors of OS (**Table 3**). For PFS, patients with PD1>50% (hazard ratio [HR] = 4.385, *P* = 0.001) or metastatic organs ≥2 (HR = 0.245, *P* = 0.04) had worse outcomes (**Table 4**). Subsequently, Kaplan-Meier survival plots were generated based on the significant prognostic factors identified in the multivariate Cox model analysis. Patients with more than two metastatic organs (10.9 vs. 20.7 months, *P* = 0.001) or PD1 expression less than 50% (13.6 vs. 21.9 months, *P*= 0.000) exhibited significantly shorter overall survival compared to their counterparts, as depicted in **Figures 5A-D**. Furthermore, the presence of two or more metastatic organs (7.3 vs. 15.2 months, *P* = 0.000) or a PD1 level exceeding 50% (9.0 vs. 16.6 months, *P* = 0.000) exhibited a significant correlation with reduced intracranial PFS, as depicted in **Figures 5A-D**).

**Table 3:** Univariate and multivariable Cox regression analysis for OS.

Characteristic	Univariate survival analyses of OS			Multivariable survival analyses of OS		
	HR	95%CI	P Value	HR	95%CI	*P* Value
Age						
< 60						
≥ 60	0.595	0.273-1.297	0.192			
Sex						
Male						
Female	0.965	0.855-10.277	0.087			
ECOG						
0–1						
2	1.643	0.556-4.850	0.920			
Smoking history						
Never						
Smoked	0.725	0.355-1.481	0.378			
Metastatic organs						
<2						
≥ 2	0.053	0.014-0.201	0.000	0.101	0.034-0.302	0.000
Number of BMs						
Single						
Multiple	0.885	0.288-2.720	0.831			
Symptomatic BMs						
No						
Yes	0.683	0.354-1.316	0.254			
LDH						
< ULN						
≥ ULN	1.383	0.679-2.834	0.396			
PD1						
>50%						
<50%	0.939	17.428-21.109	0.000	3.313	1.398-7.853	0.007

**Table 4:** Univariate and multivariable Cox regression analysis for PFS.

Characteristic	Univariate survival analyses of PFS			Multivariable survival analyses of PFS		
	HR	95%CI	P Value	HR	95%CI	*P* Value
Age						
< 60						
≥ 60	0.901	0.410-1.978	0.794			
Sex						
Male						
Female	1.922	0.615-6.008	0.261			
ECOG-PS						
0–1						
2	1.437	0.494-4.181	0.506			
Smoking history						
Never						
Smoked	1.032	0.485-2.196	0.935			
metastatic organs						
<2						
≥2	0.182	0.059-0.563	0.003	0.245	0.094-0.635	0.04
Number of BMs						
Single						
Multiple	0.853	0.290-2.511	0.773			
Symptomatic BMs						
No						
Yes	0.960	0.505-1.824	0.901			
LDH						
< ULN						
≥ ULN	1.203	0.597-2.423	0.604			
PD1						
>50%						
<50%	0.818	11.318-14.525	0.006	4.385	1.829-10.515	0.001

### Safety

An analysis of TRAEs associated with cadonilimab plus anlotinib is shown in **Table 5**. Of the 46 patients, 26 (56.5%, n=26) experienced grade 1-2 treatment-related adverse events (TRAEs), while 6 patients (13%) encountered grade 3-4 TRAEs.As a whole, hypertension accounted for 52%, hypertension accounted for 50%, and leukemia accounted for 30.4%. Among the grade 3–4 TRAEs, Hypertension accounted for the highest occurrence rate (6.5%, n =3), followed by thyroid dysfunction (4.3%, n = 2). Leukopenia, Hepatic dysfunction, and positive urinary protein were ranked third, with an occurrence rate of 2.2% (n = 1).

**Table 5:** Treatment-related adverse events.

Adverse event,N (%)	Patients (n = 46)		
	ALL	Grade 1-2	Grade 3-4
Any adverse event	32 (69.6%)	26 (56.5%)	6 (13.0%)
Thyroid dysfunction	24 (52.2%)	22 (47.8%)	2 (4.3%)
Hypertension	23 (50%)	20 (58.7%)	3 (6.5%)
Leukopenia	17 (30.4%)	16 (34.8%)	1 (2.2%)
Hepatic dysfunction	9 (19.6%)	8 (17.4%)	1 (2.2%)
Thrombocytopenia	3 (6.5%)	3 (6.5%)	0
Hand-foot skin reaction	6 (13.0%)	6 (13.0%)	0
Gastrointestinal response	3 (6.5%)	3 (6.5%)	0
Positive urinary protein	2 (4.3%)	1 (2.2%)	1 (2.2%)
Dental ulcer	1 (2.2%)	1 (2.2%)	0
Cough	1 (2.2%)	1 (2.2%)	0
Fatigue	2 (4.3%)	2 (4.3%)	0
Rash	2 (4.3%)	2 (4.3%)	0
Pneumonitis	1 (2.2%)	1 (2.2%)	0
Diarrhea	1 (2.2%)	1 (2.2%)	0

## Discussion

In the realm of small-cell lung cancer (SCLC), no parallel research has been conducted to evaluate the efficacy of Cadonilimab combined with anlotinib for brain metastases (BMs) in the central nervous system. While intracranial radiotherapy is widely employed for BM treatment, it has detrimental effects on the surrounding healthy tissues, which can impair the patient’s cognitive function, memory, and intellectual capacity. The search for novel therapeutic approaches is crucial, as the majority of SCLC patients with BMs, even after undergoing systemic radiotherapy, continue to face recurrence or develop resistance to treatment. To the best of our knowledge, this study is the first to report the use of Cadonilimab combined with anlotinib for the treatment of extensive SCLC with brain metastases (BMs) in real-world clinical practice. The use of Cadonilimab combined with anlotinib regimens provided a 19.3 months of OS and 14.2 months of PFS. This regimen showed significant efficacy within the central nervous system. We found that a metastatic organs≥ 2 and PD1>50% concentration were independent predictive factors associated with shorter OS in the studied patients. Thyroid dysfunction and Hypertension accounted for most of the treatment-related AEs in this study; these AEs were manageable, and no treatment-related deaths occurred.

In recent years, there has been significant advancement in immuno-oncology (IO) treatments, with monoclonal antibodies (mAbs) targeting programmed cell death-1 (PD-1) now recognized as the standard treatment for various types of cancer. Numerous combinations of anti-PD-1 antibodies have been explored to enhance the efficacy of PD-1 monotherapy. Promising research has shown significant improvements in the efficacy of combination therapy involving anti-cytotoxic T-lymphocyte antigen-4 (CTLA-4) and anti-PD-1 antibodies for difficult-to-treat cancer types. However, its use in kidney, gastric, and lung cancers has been restricted because of its toxic effects. Cadonilimab represents a humanized IgG1 bispecific antibody, engineered for enhanced functionality via the Akeso Tetrabody platform. Yet, it lacks the conventional Fc effector functions such as antibody-dependent cellular cytotoxicity, phagocytosis, and complement-mediated cytotoxicity. This dual-targeting agent simultaneously engages PD-1 and CTLA-4, thereby inhibiting the PD-1/PD-L1, PD-1/PD-L2, CTLA-4/B7.1, and CTLA-4/B7.2 interactions. Preliminary investigative findings in preclinical research suggest that cadonilimab’s selective accumulation within tumor tissues, in contrast to standard anti-PD-1 and anti-CTLA-4 antibodies, may contribute to an enhanced safety profile. Initial results from the phase 1 trial of cadonilimab imply that it might confer superior tolerance compared to the concurrent administration of PD-1 and CTLA-4 inhibitors. Moreover, in June 2022, cadonilimab received marketing authorization in China for the treatment of patients with relapsed or metastatic cervical cancer following progression on platinum-based chemotherapy, based on the encouraging outcomes of a pivotal phase II clinical trial. Just a few years ago, the oncology community firmly dismissed the idea of using immunotherapy to treat brain metastases. Immune checkpoint-targeting monoclonal antibodies have demonstrated significant efficacy against various types of tumors; however, individuals with brain disorders are consistently ineligible for participation in clinical trials. Treatment options for patients with brain metastases include surgical removal, whole-brain radiation therapy (WBRT), stereotactic radiosurgery (SRS), and their combinations (12). Chemotherapy is rarely utilized because of its limited ability to effectively cross the blood-brain barrier (13). Historically, individuals with BM, as well as their unfavorable prognosis, have typically been ineligible for chemotherapeutic trials. This identical situation has similarly been extended to immunotherapy involving immune checkpoint inhibitors (ICIs) in recent times (14). In the past few years, scientists have examined the connections between the immune system and the tumor microenvironment (TME) in brain metastases, leading to the recognition of the central nervous system (CNS) as a separate immunological area rather than an isolated one (15). The majority of individuals with brain metastases possess an inflamed tumor microenvironment (TME) that is invaded by tumor-infiltrating lymphocytes (TIL). These lymphocytes frequently exhibit immunosuppressive elements such as programmed death-1 (PD-1) ligand (PD-L1) (16). Recently, anti-CTLA-4, anti-PD-1, and anti-PD-L1 antibodies have emerged, which further endorse their application in immunotherapy. Moreover, these antibodies are utilized in patients with brain metastases and CNS tumors that develop in the brain. Patients diagnosed with non-small cell lung cancer and currently experiencing brain metastases were not included in the clinical trials involving ICI treatment. However, a limited number of retrospective studies have been conducted to compare the effectiveness of ICI therapy with that of alternative treatments in this specific patient population. During a phase II study, pembrolizumab demonstrated a 29.4% intracranial objective response rate (ORR) in 10 of 34 patients with PD-L1+ status. No objective response was observed in the patients without PD-L1 expression. The median overall survival for all patients was 8.9 months, with a 31% survival rate at 2 years (17). The role of nivolumab in patients with non-small cell lung cancer (NSCLC) who had asymptomatic brain metastases, either previously treated or untreated, was analyzed by combining the findings from the CheckMate 063 (phase II), 017 (phase III), and 057 (phase III) trials (18). During the assessment of patients with previously treated brain metastases at the point of overall disease progression (PD) or the latest tumor evaluation, 33% exhibited no indications of central nervous system (CNS) advancement, whereas 52% did. Nivolumab therapy demonstrated a longer median overall survival (8.4 months) in comparison to docetaxel chemotherapy. According to the Italian expanded access program, nivolumab has proven successful in the treatment of NSCLC patients with brain metastases who are either asymptomatic or have received prior treatment for brain metastases. The program reported an overall response rate (ORR) of 17% and a disease control rate (DCR) of 40% (19). Moreover, an exploratory subgroup analysis was conducted on the OAK study (20), Considering patients who have or have not previously had asymptomatic, treated brain metastases, anti-PD-L1 atezolizumab showed a favorable safety profile, and ezolizumab compared to docetaxel showed a trend in favor of a longer OS (16 versus 11.9 months). In contrast to docetaxel, atezolizumab delays the radiological identification of new symptomatic brain metastases (21).

The effectiveness of ICIs in improving survival in SCLC patients with BMs is uncertain, but ICIs have shown promise in treating other types of tumors, such as NSCLC and melanoma. The study revealed that atezolizumab did not show any survival advantage when compared to chemotherapy alone in IMpower133, which included 35 patients with SCLC and BMs. Similarly, durvalumab, another PD-L1 inhibitor, did not demonstrate any survival benefit. Nevertheless, individuals who receive serplulimab, a PD-1 inhibitor, could potentially experience advantages from ASTRUM-005 (HR, 0.61, 0.33–1.13) (22), The mPFS and mOS data of this study are much better than those of previous studies were related to cadonilimab (PD-1/CTLA-4 bispecific antibody) plus anlotinib regimen, and also related to the physical condition of enrolled patients and the underlying factors of metastatic tumor. Clinical studies of anti-PD-1/PD-L1 antibodies in combination with antiangiogenic drugs have shown good efficacy and manageable safety in the treatment of various solid tumors, recent studies (11) showed the superiority of immunization with anlotinib, specifically cadonilimab (PD-1/CTLA-4 bispeci-c antibody) plus anlotinib, in lung cancer. In a previous study, anlotinib was shown to be successful in treating SCLC patients with BMs. It significantly enhanced PFS (3.8 vs 0.8 months, *P* = 0.001) and OS (6.1 vs 2.6 months, *P* = 0.006) when used as a third-line or subsequent treatment, surpassing the effectiveness of a placebo (7). The clinical potential of using Anlotinib in combination with ICIs for treating patients Several clinical factors have a negative impact on the outcomes of patients with BMs who receive ICIs.There was a significant association between poor survival rates and patients with more than two metastatic organs (23–26). As found in our study, high disease burden and PD1>50% were independent negative predictors of both OS and PFS. Moreover, prior research has indicated that increased LDH levels can have a detrimental impact on the effectiveness of immunotherapy when it induces T-cell immunosuppression in cancer (27). Ankush et al. discovered that individuals diagnosed with melanoma and experiencing BMs who underwent treatment with ICIs exhibited reduced rates of survival when LDH levels were elevated (HR, 2.45, 1.16–5.16, *P* = 0.019) (27). Additionally, patients with multiple BMs have a more unfavorable prognosis than those with only one BM (27–31). However, our study did not yield similar findings. The variation in tumor immunogenicity among different tumor types or the absence of a control group in the study, along with the small sample size, may explain this.The results of our study indicate that the use of Cadonilimab combined with anlotinib regimens as Second or post-line therapy in extensive-stage SCLC is effective and feasible in clinical practice.

The combination of Cadonilimab (a bispecific antibody targeting PD-1 and CTLA-4) and Anlotinib (a multi-target tyrosine kinase inhibitor) demonstrated a clinically manageable safety profile in patients with extensive-stage small cell lung cancer (SCLC) and brain metastases (BMs). Treatment-related adverse events (TRAEs) of any grade occurred in 69.6% of patients, with grade 3-4 TRAEs observed in 13.0% of cases, consistent with prior reports on immune checkpoint inhibitor (ICI)-Anlotinib combinations (28–32). Notably, the incidence of grade 3-4 hypertension (6.5%) and thyroid dysfunction (4.3%) exceeded rates observed in advanced non-small cell lung cancer (NSCLC) cohorts treated with PD-1 inhibitors plus Anlotinib (grade 3-4 TRAEs: ~40%) (24). This discrepancy may arise from tumor-specific pathophysiological factors, such as SCLC-associated cachexia and aggressive disease biology, or cumulative toxicities from prior therapies (31, 32).

Our study had several limitations. as a retrospective study, this study has inherent limitations associated with retrospective studies. Additionally, the potential power of the study could be limited because of its small size and the absence of a control group. Furthermore, Although our study’s patients were drawn from single institutions, The ethnic and geographic characteristics of this study’s patients were homogeneous. Although the regimens of the combination treatments were not all the same as immune checkpoint inhibitors plus anlotinib in small cell lung cancer, neither the efficacy nor safety was negatively affected. Based on the results of our study, further clinical studies should focus on the effectiveness and safety of Cadonilimab combined with Anlotinib in extensivestage SCLC patients of Metastatic organs≥ 2, PD1>50%, ethnic groups other than East Asian, and those from different geographic areas.

## Conclusions

Cadonilimab combined with Anlotinib are feasible for extensive-stage SCLC patients with central nervous system for treating BMs considering their efficacy and safety. Metastatic organs≥ 2 and PD1>50% were associated with unfavorable outcomes, and further therapeutic strategies may be needed for these patients.

## Data Availability

The datasets presented in this study can be found in online repositories. The names of the repository/repositories and accession number(s) can be found in the article/supplementary material.

## References

[1] Rudin CM Brambilla E Faivre-Finn C Sage J. Small-cell lung cancer. Nat Rev Dis Primers. (2021) 7:3. doi: 10.1038/s41572-020-00235-0, PMID: 33446664 PMC8177722

[2] Rusthoven CG Yamamoto M Bernhardt D Smith DE Gao D Serizawa T. Evaluation of first-line radiosurgery vs whole-brain radiotherapy for small cell lung cancer brain metastases. JAMA Oncol. (2020) 6:1028–37. doi: 10.1001/jamaoncol.2020.1271, PMID: 32496550 PMC7273318

[3] Rittberg R Banerji S Kim JO Rathod S Dawe DE. Treatment and prevention of brain metastases in small cell lung cancer. Am J Clin Oncol. (2021) 44:629–38. doi: 10.1097/COC.0000000000000867, PMID: 34628433

[4] Liu SV Reck M Mansfield AS Mok T Scherpereel A Reinmuth N. updated overall sur- vival and pd-l1 subgroup analysis of patients with extensive-stage small-cell lung cancer treated with atezolizumab, carboplatin, and etoposide (IMpower133). J Clin Oncol. (2021) 39:619–30. doi: 10.1200/JCO.20.01055, PMID: 33439693 PMC8078320

[5] Rudin CM Awad MM Navarro A Gottfried M Peters S Csőszi T. Pembrolizumab or placebo plus etoposide and platinum as first-line therapy for extensive-stage small-cell lung cancer: randomized, double-blind, phase III KEYNOTE-604 study. J Clin Oncol. (2020) 38:2369–79. doi: 10.1200/JCO.20.00793, PMID: 32468956 PMC7474472

[6] Friebel J Moritz E Witkowski M Jakobs K Strässler E Dörner A. Pleiotropic effects of the protease-activated receptor 1 (PAR1) inhibitor, vorapaxar, on atherosclerosis and vascular inflammation. Cells. (2021) 10:3517. doi: 10.3390/cells10123517, PMID: 34944024 PMC8700178

[7] Cheng Y Wang Q Li K Shi J Han B Wu L. P2.12-26 the impact of anlo- tinib for relapsed sclc patients with brain metastases: a subgroup analysis of ALTER 1202. J Thorac Oncol. (2019) 14:S823–4. doi: 10.1016/j.jtho.2019.08.1771

[8] Liu S Qin T Liu Z Wang J Jia Y Feng Y. Anlotinib alters tumor immune microenvironment by downregulating PD-L1 expression on vas- cular endothelial cells. Cell Death Dis. (2020) 11:309. doi: 10.1038/s41419-020-2511-3, PMID: 32366856 PMC7198575

[9] Morse MA Overman MJ Hartman L Khoukaz T Brutcher E Lenz HJ. Safety of nivolumab plus low-dose ipilimumab in previously treated microsatellite instability-high/mismatch repair-deficient metastatic colorectal cancer. Oncologist. (2019) 24(11):1453–61. doi: 10.1634/theoncologist.2019-0129, PMID: 31147488 PMC6853093

[10] Motzer RJ Rini BI McDermott DF Arén Frontera O Hammers HJ Carducci MA. Nivolumab plus ipilimumab versus sunitinib in first-line treatment for advanced renal cell carcinoma: extended follow-up of efficacy and safety results from a randomised, controlled, phase 3 trial. Lancet Oncol. (2019) 20:1370. doi: 10.1016/S1470-2045(19)30413-9, PMID: 31427204 PMC7497870

[11] Chen B Yao W Li X Lin G Chu Q Liu H. A phase Ib/II study of cadonilimab (PD-1/CTLA-4 bispecific antibody) plus anlotinib as first-line treatment in patients with advanced non-small cell lung cancer. Br J Cancer. (2024) 130:450–6. doi: 10.1038/s41416-023-02519-0, PMID: 38110665 PMC10844309

[12] Chamberlain MC Baik CS Gadi VK Bhatia S Chow LQ. Systemic therapy of brain metastases: non-small cell lung cancer, breast cancer, and melanoma. Neuro-Oncology. (2017) 19:i1–i24. doi: 10.1093/neuonc/now197, PMID: 28031389 PMC5193029

[13] Ostrom QT Wright CH Barnholtz-Sloan JS. Brain metastases: epidemiology. Handb Clin Neurol. (2018) 149:27–42. doi: 10.1016/B978-0-12-811161-1.00002-5, PMID: 29307358

[14] Kaufman HL Atkins MB Subedi P Wu J Chambers J Mattingly TJ. The promise of Immuno-oncology implications for defining the value of cancer treatment. J Immunother Cancer. (2019) 7:129. doi: 10.1186/s40425-019-0594-0, PMID: 31101066 PMC6525438

[15] Quail DF Joyce JA. The microenvironmental landscape of brain tumors. Cancer Cell. (2017) 31:326–41. doi: 10.1016/j.ccell.2017.02.009, PMID: 28292436 PMC5424263

[16] Berghoff AS Venur VA Preusser M Ahluwalia MS. Immune checkpoint inhibitors in brain metastases: from biology to treatment. Am Soc Clin Oncol Educ Book. (2016) 35:e116–22. doi: 10.1200/EDBK_100005, PMID: 27249713

[17] Goldberg SB Gettinger SN Mahajan A Herbst RS Chiang AC Lilenbaum R Lin G Chu Q Liu H. Durability of brain metastasis response and overall survival in patients with non-small cell lung cancer (NSCLC) treated with pembrolizumab. J Clin Oncol. (2018) 36:2009. doi: 10.1200/JCO.2018.36.15_suppl.2009 29787359

[18] Goldman JW Crinò L Vokes EE Holgado E Reckamp KL Pluzanski A. Nivolumab in patients with advanced NSCLC and central nervous system metastases. J Clin Oncol. (2016) 34:9038. doi: 10.1200/JCO.2016.34.15_suppl.9038

[19] Crinò L Bronte G Bidoli P Cravero P Minenza E Cortesi E. Nivolumab and brain metastases in patients with advanced non-squamous non-small cell lung cancer. Lung Cancer. (2019) 129:35–40. doi: 10.1016/j.lungcan.2018.12.025, PMID: 30797489

[20] Rittmeyer A Barlesi F Waterkamp D Park K Ciardiello F von Pawel J. Atezolizumab versus docetaxel in patients with previously treated non-small-cell lung cancer (OAK): a phase 3, open-label, multicentre randomised controlled trial. Lancet. (2017) 389:255–65. doi: 10.1016/S0140-6736(16)32517-X, PMID: 27979383 PMC6886121

[21] Gadgeel SM Lukas RV Goldschmidt J Conkling P Park K Cortinovis D. Atezolizumab in patients with advanced non-small cell lung cancer and history of asymptomatic, treated brain metastases: exploratory analyses of the phase III OAK study. Lung Cancer. (2019) 128:105–12. doi: 10.1016/j.lungcan.2018.12.017, PMID: 30642441

[22] Cheng Y Han L Wu L Conkling P Park K Cortinovis D. Serplulimab, a novel anti-PD-1 antibody, plus chemotherapy versus chemotherapy alone as first-line treatment for extensive-stage small-cell lung cancer: Aninternational randomized phase 3 study. J Clin Oncol. (2022) 40:8505. doi: 10.1200/JCO.2022.40.16_suppl.8505

[23] Dou XJ Ma RY Ren DW Liu Q Yan P. Effectiveness and safety of anlotinib combined with PD-1 blockades in patients with previously immunotherapy treated advanced non-small cell lung cancer: A retrospective exploratory study. Lung Cancer (Auckl). (2024) 15:29–40. doi: 10.2147/LCTT.S444884, PMID: 38560413 PMC10979677

[24] Wu Y Zhang T Liu Y Wang J Bi N. Anlotinib combined with durvalumab in a patient with recurrent multifocal brain metastases of small cell lung cancer after definitive concurrent chemoradiotherapy and palliative radiotherapy of the lung and brain: a case report. Ann Palliat Med. (2021) 12(12):3707. doi: 10.21037/apm-20-2390, PMID: 33725780

[25] Skribek M Rounis K Makrakis D Wang J Bi N. Outcome of patients with NSCLC and brain metastases treated with immune checkpoint inhibitors in a “real-life” setting. Cancers. (2020) 10(2):2379–86. doi: 10.3390/cancers12123707, PMID: 33321730 PMC7764720

[26] Hendriks L Henon C Auclin E Agelaki S Mavroudis D De Petris L. Outcome of patients with non-small cell lung cancer and brain metastases treated with checkpoint inhibitors. J Thorac Oncol. (2019) 14:1244–54. doi: 10.1016/j.jtho.2019.02.009, PMID: 30780002

[27] Ding J Karp JE Emadi A. Elevated lactate dehydrogenase (LDH) can be a marker of immune suppression in cancer: interplay between hematologic and solid neoplastic clones and their microenvironments. Cancer Biomark. (2017) 19:353–63. doi: 10.3233/CBM-160336, PMID: 28582845 PMC13020749

[28] Mezquita L Auclin E Ferrara R Charrier M Remon J Planchard D. Association of the lung immune prognostic index with immune checkpoint inhibitor outcomes in patients with advanced non-small cell lung cancer. JAMA Oncol. (2018) 4:351–7. doi: 10.1001/jamaoncol.2017.4771, PMID: 29327044 PMC5885829

[29] Hazuka MB Burleson WD Stroud DN Leonard CE Lillehei KO Kinzie JJ. Multiple brain metastases are associated with poor survival in patients treated with surgery and radiotherapy. J Clin Oncol. (1993) 11:369–73. doi: 10.1200/JCO.1993.11.2.369, PMID: 8426215

[30] Shi Y Ji M Jiang Y Yin R Wang Z Li H. A cohort study of the efficacy and safety of immune checkpoint inhibitors plus Anlotinib versus immune checkpoint inhibitors alone as the treatment of advanced non-small cell lung cancer in the real world. Transl Lung Cancer Res. (2022) 11:1051–68. doi: 10.21037/tlcr-22-350, PMID: 35832459 PMC9271442

[31] Feng Y Tang L Wang H Liu Y Yang S Lin L. Immune checkpoint inhibitors combined with angiogenic inhibitors in the treatment of locally advanced or metastatic lung adenocarcinoma patients. Cancer Immunol Immunother. (2022) 72(2):449–59. doi: 10.1007/s00262-022-03251-z, PMID: 35934742 PMC10991245

[32] Wang X Guo G Guan H Yu Y Lu J Yu J. Challenges and potential of PD-1/PD-L1 checkpoint blockade immunotherapy for glioblastoma. J Exp Clin Cancer Res. (2019) 38:87. doi: 10.1186/s13046-019-1085-3, PMID: 30777100 PMC6380009

